# Can Kratom (*Mitragyna speciosa*) Alleviate COVID-19 Pain? A Case Study

**DOI:** 10.3389/fpsyt.2020.594816

**Published:** 2020-11-19

**Authors:** Antonio Metastasio, Elisabeth Prevete, Darshan Singh, Oliver Grundmann, Walter C. Prozialeck, Charles Veltri, Giuseppe Bersani, Ornella Corazza

**Affiliations:** ^1^School of Life and Medical Sciences, University of Hertfordshire, Hatfield, United Kingdom; ^2^NHS Camden and Islington Trust, London, United Kingdom; ^3^Department of Medico-Surgical Sciences and Biotechnologies, Sapienza University of Rome, Rome, Italy; ^4^Centre for Drug Research, Universiti Sains Malaysia, Minden, Malaysia; ^5^Department of Medicinal Chemistry, College of Pharmacy, University of Florida, Gainesville, FL, United States; ^6^Department of Pharmacology, Midwestern University, Downers Grove, IL, United States; ^7^Department of Pharmaceutical Sciences, Midwestern University, Glendale, AZ, United States

**Keywords:** COVID-19, kratom, opioid, stimulants, new psychoactive substances, new treatments, long covid

## Abstract

Among the symptoms of COVID-19 fever, general malaise, pain and aches, myalgia, fatigue, and headache can affect the quality of life of patients, even after the end of the acute phase of the infection and can be long lasting. The current treatment of these symptoms, also because COVID-19 patients have been asked not to use non-steroidal anti-inflammatory drugs (NSAIDs), in particular ibuprofen are often unsatisfactory. Among the above mentioned symptoms malaise and fatigue seem the most difficult to treat. In this case report we describe the use of kratom (*Mitragyna speciosa*) by a patient with confirmed COVID-19 infection. What we observed was a fast and sustained relieve of the above mentioned symptoms.

## Background

Viral infections, including the current COVID-19 pandemic, are often associated with fever, general malaise, pain, and aches (1, 2). Of these, fever (98%), cough (76%), dyspnoea (55%), myalgia or fatigue (44%), headache (8%), and haemoptysis (5%) are commonly noted (2). These infections, therefore, even in the milder and non-life threatening forms, can significantly affect the quality of life. Among the various peculiarities of the COVID-19 infection concerns have been raised about the use of non-steroidal anti-inflammatory drugs (NSAIDs), in particular ibuprofen, which at first seemed to worsen the illness, although further studies have disproved this concern (3). As a consequence, several regulatory agencies, including the European Medicines Agency (EMA), at first expressed concerns about its use, as it may deprive patients of an effective treatment for fever and pain with the exception of paracetamol/acetaminophen (3). The debate about NSAID safety is still open. At the beginning of the pandemic, ibuprofen was hypothesized to increase the risk of severe adverse events in COVID-19 patients and a link between NSAIDs and angiotensin-converting enzyme (ACE) 2 receptors upregulation was suggested to be involved (4). Further, Micallef et al. (5) reported that some preclinical evidences, such as immunomodulatory effects or antibiotics efficacy reduction, would support a possible link between NSAIDs and complications in COVID-19 patients. However, data about NSAIDs use in COVID-19 is still inconsistent. In fact, some authors suggested that NSAIDs should be avoided in COVID-19 (6, 7) and others reported that NSAID use has been associated with worse outcomes (4). At the same time, other authors highlighted that evidence about the worsening of COVID-19 symptoms by ibuprofen is lacking (8, 9) or only suggested to be prudent in the prescription (10).

Up to date it is possible to say that there is not an unique point of view and the controversial NSAIDs use in COVID-19 is still discussed (8), with recently a positive insight on ibuprofen in COVID-19 disease (11). As consequence there has been a drop in ibuprofen sales (as reported by Glaxo Smith Kline–GSK) in the second quarter of 2020 (12). Considering the burden that COVID-19 infection is imposing to the world population (both in the acute phase and in the so called “*long COVID*”) we thought important, therefore, to consider also other treatments that could expand our pharmaceutical armamentarium that could alleviate the symptoms of COVID-19 Infection.

In this case report we describe the use of kratom (*Mitragyna speciosa*), a plant used in traditional medicine in South-East Asia for its therapeutic benefits in self-managing opioid dependence and withdrawal, psychological disorders (e.g., anxiety and depression), and chronic pain (13, 14), and to successfully alleviate COVID-19 related symptoms. Kratom contains more than 40 alkaloids (15, 16), though the majority of its pharmacological properties appear to be related to two of the active compounds: mitragynine and its metabolite 7-hydroxymitragynine (17–19). Kratom is reported to have opioid and non-opioid like effects. In traditional settings in Malaysia and Thailand, rural folks traditionally use kratom as a remedy to treat common health maladies, and kratom consumption practice/tradition do not seem to cause any significant health problems (20–22).

The results of several anonymous online surveys have indicated that the use of kratom products may be useful for the self-treatment of acute and chronic pain (23–25), and in fact, its use is only self-reported to be associated with few adverse effects. Findings from a recent clinical trial confirmed the analgesic properties of kratom in healthy volunteers lasting for approximately 2 h with average blood concentrations of mitragynine at 2,000 ng/mL (26). These results suggest that kratom has the potential to be used as a centrally acting herbal analgesic.

Although kratom is reported to be used as a safe substitute to opioids in self-managing pain, dependence and withdrawal (23–25), it is not free from adverse effects and risks. Kratom dependence has been reported if the product is used in larger quantity over a prolonged period, and negative effects such as sleep problems, depressed mood, diarrhea, and flu-like symptoms including muscle and joint pain can develop with sudden withdrawal (27). Fatalities involving kratom are rare and, autopsy findings indicate that in such instances kratom is concurrently used with illicit substances or anti-depressants, and not kratom *per se*, or the user had an underlying health condition (28, 29). So far, there have been no reports specifically on fatal kratom overdose incidences (30).

We are aware, however, that there is a lack of robust data about kratom efficacy in humans, to the best of our knowledge there is only one randomized controlled trial that would give some support to kratom's therapeutic potential in pain. Most of the information available today are the results of surveys and of retrospective studies, in which users claim Kratom's efficacy in treating acute and chronic pain of different etiologies (23, 24). Other conditions that appear to benefit from kratom are headache (24, 25), back, neck and muscle pain (24, 25), fibromyalgia, arthritis (including autoimmune ones like rheumatoid arthritis), autoimmune disorders like multiple sclerosis (13, 25), and other severe conditions like cancer and chronic inflammatory diseases (25). Some autheors have therefore speculated that kratom has a role in the Central Nervous System (CNS) but also as anti-inflammatory (31–33), muscle relaxant (34).

Despite the potential therapeutic benefit, kratom has also severe side effects, that should be always considered when suggesting or only considering a treatment with kratom (35). Among the most severe side effects have been described kratom associated hepatitis (36–39), seizures and coma (40, 41), hypogonadism (42), hypothyroidism (43), posterior reversible leukoencephalopathy (44), fatalities (29, 45) and overdoses (46, 47). It is important to underlie, however that most of these events were described mainly in the US and Europe (where Kratom was recently introduced), with a majority of the reported deaths involving the presence of other substances (29), such as benzodiazepine, opioids, antidepressant or antipsychotic agents, alcohol or other substances, e.g., Datura stramonium, cannabinoids, amphetamines (40, 45, 48–51), and other contaminant such as O-desmethyltramadol (52).

There is growing evidence, however, that kratom is safer if used as pure kratom products or brewed herbal decoction in small doses and for a limited period of time. It should be avoided the consumption of large amounts (more than 15 grams per dose) and high frequencies (more than 3 times/day for extended periods of time) because the risk of developing dependence. Several cases have been reported in both Western (53–55), including cases of neonatal abstinence syndrome (56, 57), and Eastern countries (27, 58, 59), where those who used kratom for a long time experienced both physical (e.g., constipation) and psychological (e.g., anxiety) withdrawal symptoms. More recently an article have been published by Muller et al. (60) in which an individual self-prescribing kratom for pain treatment reported an escalation of the dosage needed and eventually developed a dependence.

Considering the conflicting evidence and the paucity of randomized control studies the balance between kratom benefits and risks is not clear yet, but some data suggested that kratom may cause less issues compared to opioids as well as retrospective data showed that kratom reduced the prevalence opioid adverse effects in users (24) and among illicit opioid users (61).

## Case Report

### Case Presentation

The subject of this report is a 29 year old male, US citizen of Palestinian descent, who works full-time as a biomedical research technician. His health history is unremarkable, except for the fact that at age 16 he was diagnosed with ulcerative colitis and primary sclerosing cholangitis. Since then, he has been treated successfully with mesalamine (1.2 g, 2 times per day), azathioprine (50 mg, 3 times per day), and ursidiol (300 mg, 2 times per day). The subject has been able to live an active lifestyle and participate in a variety of sports including running, weightlifting, basketball, and baseball. The subject denies any history of smoking or use of alcohol, opioids, or illicit drugs. On April 22, 2020, the subject's father, who lives in the same house as the subject, and works for a major shipping company, was diagnosed with COVID-19. This was about 2 months after the first case of COVID had been confirmed in his state of residence, which was one of the early active zones of COVID-19 transmission in the US. On April 25, the patient began to experience general malaise and fatigue. Over the next 24 h, the symptoms worsened to include severe fatigue and weakness, loss of appetite, tiredness, slight dry cough, body aches, muscle pain, loss of taste and smell, sore throat and fever. The patient was then seen by his general practice physician. Vital signs at the time of examination were BP 110/72, pulse 97 BPM, respiration 14 per minute, oxygen saturation 98%, and body temperature 101.7°F. The patient was given a naso-pharyngeal swab, real-time RT-PCR test [COBAS (R) SARS-COV-@ test, Labcorp Laboratories, South Bend, IN] that confirmed a diagnosis of COVID-19. In compliance with standard medical practice standards, the patient was ordered to self-isolate and to start a 5-day course of azithromycin (250 mg, daily), and to also take 1 g of paracetamol (acetaminophen) every 6 h for treatment of pain and fever. Despite good adherence to the recommended treatment, the symptoms other than fever, did not improve, and he also started to feel depressed, demotivated, and spend long periods in bed. During this period, the patient experienced ongoing generalized myalgia and musculoskeletal pain. He described the pain as persistent and relatively severe (rated 7 on a scale of 1–10). Because of this discomfort, after 4 days the patient decided to consume kratom to relieve his symptoms. According to the patient, he had first used kratom 14 months earlier before his COVID-19 infection. He used kratom sporadically (no more than 4–5 times in total) as a cognitive enhancer and not to self-treat pain.

### Treatment

The patient decided to take 2.5 gms (or grams) of green kratom (as ground leaf powder suspended in water). The product was purchased at a local shop in April 2020 sold under the name “Green Bali.” After 30 min, he noticed a significant improvement in the intensity of the physical symptoms (mainly pain and fatigue), and within 60 min he felt a sensation of mild euphoria and well-being that lasted for about 5 h. After 6 h following consumption the effects of kratom wore off, and the patient administered another dose. He used kratom three times a day continuously for 3 days (for a total of 9 doses of 2.75 g each) with significant benefit.

### Outcome

When asked to score from 0 to 100% the improvement that kratom had on COVID-19 symptoms: fatigue and weakness (80% improvement), tiredness (70% improvement), body aches (80% improvement), muscle pain (90% improvement, “much better than paracetamol/acetaminophen”). The kratom did not seem to have an impact on: fever, cough, or sore throat. The patient also stated: “I didn't have anxiety or any psychological symptoms. For me, kratom mainly gave improvement in physical reaction.” “It also elevated my mood and made me feel less miserable, to the point where I was able to get out of bed, shower, look at work emails without feeling completely exhausted and drained”; “Kratom helped me more than antibiotic”; “I slept better, I essentially fell asleep immediately. Without kratom, sleep was not nice, with kratom less wake ups, about 6 h.” Over the next 2 weeks the patient's symptoms gradually subsided and on May 13 he had a televisit with his physician and a follow-up swab test that was negative for COVID-19. The subject was able to end his quarantine and return to work in early June. In a follow-up interview with us, the patient reported that he did not experience any side effects from using kratom, except for a very bad taste when swallowing it. The patient was also able to discontinue kratom use immediately without any evidence of physical or psychological withdrawal symptoms. The patient also informed us that he still had some of the kratom product that he had taken and he agreed to provide us with a sample for chemical analysis.

### Kratom Sample Analysis

An established quantitative liquid chromatography mass spectrometry method was conducted (62) and found that the sample obtained from the patient is kratom due to the presence of mitragynine (102 mg/g kratom powder) and 7-hydroxymitragynine (0.8 mg/g kratom powder). The extracted kratom sample was analyzed for the presence of 13 opioids and 8 benzodiazepines by comparing the chromatograms to those of the reference mixtures Pain Management Multi-component Opiate Mixture-13 solution and Benzodiazepine Multi-component Mixture-8 solution. These data suggest the sample was not fortified with 7-hydroxymitragynine and there was also no evidence of adulteration with opioids or common benzodiazepines in the sample.

## Discussion

To the best of our knowledge, this is the first case report that aims to highlight the use of kratom in alleviating COVID-19 infection related symptoms, and pain. Our findings show that short-term kratom use has the potential to alleviate COVID-19 infection symptoms, primarily pain, and did not seem to cause any physical and psychological withdrawal symptoms when kratom was discontinued after short-term use.

Kratom is an evergreen plant indigenous to Southeast Asia. Historically, kratom is a widely used folk remedy or traditional medicine. Kratom prominence grew a decade ago in Europe and the US, when it was chiefly used for its unique medicinal properties in self-managing pain, infections, opioid dependence and withdrawal (25, 63).

The antinociceptive action depends on mitragynine pharmacology: the compound acts as a partial G-protein biased agonist of mu opioid receptors (64, 65), and also as an agonist at other receptors (serotonin, adenosine-2a, dopamine-2, postsynaptic alpha-2 adrenergic) (17, 66). The antinociceptive effects of mitragynine have been studied in animal models (18, 67, 68), and human data, derived mainly from surveys or retrospective studies in users, clearly shows that kratom is used for pain relief and to improve mood (23, 24). The exact dose-response relationship is still unknown, but an average daily consumption of 76.3–114.8 mg of mitragynine (equivalent to 3.5 glasses of kratom tea/ juice) seems to be well-tolerated among users in traditional settings (69).

COVID-19 is a global emergency, and most of the clinical trials and research are dedicated to find effective treatments against the virus and the consequences of the infection. However, like many other viral and bacterial infections, COVID-19 infection is also associated with pain, aches and malaise and usually has a negative impact on the quality of life of patients. The current treatment for these symptoms is based mainly on paracetamol and/or NSAIDs. These compounds, however, are not always effective or sometimes should be avoided. It is necessary therefore to consider alternative and more effective treatments that can provide immediate reprieve from COVID-19 infection.

As far as we know, this is the first case report that aims to indicate the potential benefit of using kratom to mitigate COVID-19 related symptoms, as well as pain. Previous case reports mainly reported about the negative effects of kratom consumption that were linked to adverse events such as dependence/withdrawal syndrome, hepatic toxicity, seizures (35, 40), and fatalities (29). A majority of the reported cases involve other substances that cast doubt on the causative contribution of kratom to the adverse outcome. However, the consumption of large amounts (more than 15 g per dose) and high frequencies (more than 3 times/day for extended periods of time) of kratom is ill advised, and can increase the risk of adverse effects. Adverse effects are rarely observed with the consumption of pure kratom products or brewed herbal decoction in different doses and frequencies among users in traditional settings. Though findings from numerous studies continue to support kratom's therapeutic potential chiefly for pain relieve, at this juncture, there is no solid scientific evidences to prove its utility. More controlled clinical studies are needed to identify the pharmacological properties, safety of kratom doses, and its efficacy with the current standard treatment for pain relieve.

We think there is a promising scope for future studies in the field. However, we believe that there is a need for a series of clinical trials to identify the safe dosage and pharmacology of mitragynine, monitor, and identify potential side effects of long-term kratom use, and eventually consider a double blind randomized clinical trial to compare its efficacy with the present standard pain relieve treatment.

## Data Availability Statement

The raw data supporting the conclusions of this article will be made available by the authors, without undue reservation.

## Ethics Statement

Written informed consent was obtained from the individual(s) for the publication of any potentially identifiable images or data included in this article.

## Author's Note

We confirm that this work is original and has not been published elsewhere, nor it is currently under consideration for publication elsewhere. It is the output of a collaborative effort among the School of Life and Medical Sciences—University of Hertfordshire (United Kingdom), NHS—Camden and Islington Trust (United Kingdom), Sapienza University of Rome (Italy), Center for Drug Research—Universiti Sains Malaysia (Malaysia), Department of Medicinal Chemistry, College of Pharmacy—University of Florida (United States), Department of Pharmacology, Midwestern University (United States), and Department of Pharmaceutical Sciences—Midwestern University (United States). This publication arises from collaborative activities and staff exchanges among collaborating institutions.

## Author Contributions

AM prepared the original first draft of the manuscript and interviewed the patient with EP and OC. WCP recruited the patient. EP, DS, GB, OG, and WCP contributed to the literature review and the case study analysis. CV carried out the toxicological analysis. OC coordinated all the activities and the preparation of the manuscript. All authors collaborate to the manuscript writing — review and editing.

## Conflict of Interest

The authors declare that the research was conducted in the absence of any commercial or financial relationships that could be construed as a potential conflict of interest.
